# 2117. Outcomes and Risk Factors Associated with Development of Pneumonia in Lung Transplant Recipients in the Year After Transplant

**DOI:** 10.1093/ofid/ofac492.1738

**Published:** 2022-12-15

**Authors:** Jason C Gallagher, Torey Roesch, Julie Giurintano, Abby Atwater, Anya Dinh, Liesel Groninger, Julie Hoang, Jennifer Shif, Erin Tanasy, Jamie Wagner, Maria Bandres, Jacqueline Burnell

**Affiliations:** Temple University, Philadelphia, Pennsylvania; Temple University Hospital, Moorestown, New Jersey; Lewis Katz School of Medicine at Temple University, Philadelphia, Pennsylvania; Temple University School of Pharmacy, Glenside, Pennsylvania; Temple University - School of Pharmacy, Philadelphia, Pennsylvania; Temple University School of Pharmacy, Glenside, Pennsylvania; Temple University School of Pharmacy, Glenside, Pennsylvania; Temple University School of Pharmacy, Glenside, Pennsylvania; Temple University, Philadelphia, Pennsylvania; University of Mississippi School of Pharmacy, Jackson, Mississippi; Conway Medical Center, Conway, South Carolina; Temple University, Philadelphia, Pennsylvania

## Abstract

**Background:**

Lung transplant patients (LTPs) experience considerable infection-related morbidity and mortality, including from pneumonia. Our institution performs the highest volume of lung transplants in the US, providing an opportunity to describe the scope and impact of pneumonia in LTPs.

**Methods:**

We conducted a retrospective cohort study of all patients who received a lung transplant at our institution from late 2017 to early 2020. Patient records were reviewed from the pre-transplant period to 1-year after transplant for data pertinent to comorbidities, transplantation characteristics and complications, donor organ cultures, immunosuppression, prophylactic and therapeutic antibiotic regimens, pathogens, and outcomes. Cases of pneumonia were reviewed using standard criteria. The primary outcome was 1-year survival.

**Results:**

320 patients received lung transplants and 121(38%) developed pneumonia. Characteristics are in Table 1.

Pneumonia developed a median of 66 days after transplant (IQR 15-142). Most frequent pathogens ( >10 cases) were *P. aeruginosa*, *S. aureus*, and *K. pneumoniae*. The most common viral cause was RSV (5 cases). Patients with pneumonia had significantly lower 1-year survival rates than those without (100/120, 83.3% vs 186/199, 93.5%; p=0.004). Number of readmissions were higher in patients who had pneumonia (mean 3 ± 2.58 vs 2.04 + 3.18, p< 0.001). Bronchial stents (p< 0.001), past medical history of atrial fibrillation (0.003), and donor lung culture positive for *E. coli* (0.007) were independent risk factors for the development of pneumonia. Prophylaxis with piperacillin/tazobactam (0.032) prophylaxis with ciprofloxacin (0.008) were protective.

**Figure:**
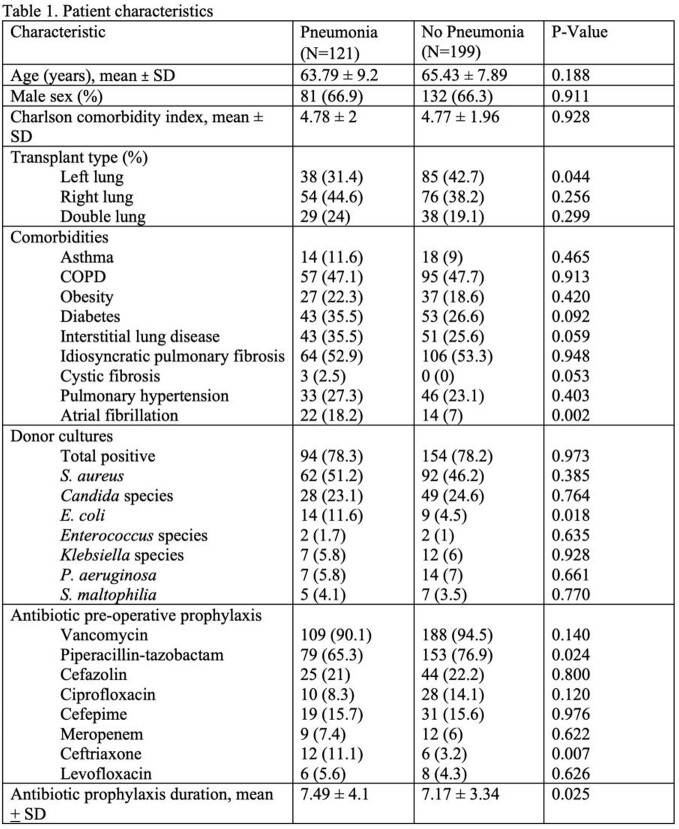

**Conclusion:**

Pneumonia occurred within the first year of transplant in 38% of LTPs and was associated with lower survival.

**Disclosures:**

**Jason C. Gallagher, PharmD**, Merck: Advisor/Consultant|Merck: Grant/Research Support|Qpex: Advisor/Consultant|Shionogi: Advisor/Consultant|Spero: Advisor/Consultant.

